# Transplant-associated thrombotic microangiopathy: theoretical considerations and a practical approach to an unrefined diagnosis

**DOI:** 10.1038/s41409-021-01283-0

**Published:** 2021-04-19

**Authors:** Joanna A. Young, Christopher R. Pallas, Mary Ann Knovich

**Affiliations:** Levine Cancer Institute, Atrium Health, Charlotte, NC USA

**Keywords:** Allotransplantation, Autotransplantation, Thrombocytopaenic purpura, Haematological diseases, Bone marrow transplantation

## Abstract

Transplant-associated thrombotic microangiopathy (TA-TMA) is an increasingly recognized complication of hematopoietic stem cell transplant (HSCT) with high morbidity and mortality. The triad of endothelial cell activation, complement dysregulation, and microvascular hemolytic anemia has the potential to cause end organ dysfunction, multiple organ dysfunction syndrome and death, but clinical features mimic other disorders following HSCT, delaying diagnosis. Recent advances have implicated complement as a major contributor and the therapeutic potential of complement inhibition has been explored. Eculizumab has emerged as an effective therapy and narsoplimab (OMS721) has been granted priority review by the FDA. Large studies performed mostly in pediatric patients suggest that earlier recognition and treatment may lead to improved outcomes. Here we present a clinically focused summary of recently published literature and propose a diagnostic and treatment algorithm.

## Introduction

Thrombotic microangiopathy is a well-recognized complication of hematopoietic stem cell transplant (HSCT), however, diagnosis can be delayed and confounded by expected cytopenias and end organ toxicities. Transplant-related factors prompt endothelial cell activation, complement dysregulation, and microvascular hemolytic anemia that can lead to end organ dysfunction and even death. Transplant-associated thrombotic microangiopathy (TA-TMA) resides within a spectrum of transplant-associated endothelial cell activation syndromes, including capillary leak syndrome, engraftment syndrome, and idiopathic pneumonia syndrome [1]. Whether hepatic veno-occlusive disease/sinusoidal obstructive syndrome (VOD/SOS) should be included within this spectrum is debated. Some exclude VOD/SOS because pathogenesis is not solely endothelial mediated. Others argue that the historical classification remains valuable [2]. The gold standard for diagnosis of TA-TMA is based on characteristic histologic findings, although bleeding risk often precludes tissue diagnosis. Due to the lack of a consensus definition for TA-TMA, the syndrome’s incidence and impact is difficult to quantify [2–8]. However, TA-TMA has a consistently reported adverse impact on non-relapse mortality (NRM) and overall survival (OS) [3–5], and the clinical presentation ranges from self-limited disease to multi-organ dysfunction and death. It impacts the kidneys, gastrointestinal tract, central nervous system, heart, lungs, and serosal surfaces [6, 7]. Treatments with wide ranging mechanisms have been implemented with variable efficacy and survival benefit. Recent focus on terminal complement blockade with eculizumab has emerged as a widely accepted therapy [8–11]. Narsoplimab (OMS721) was recently granted FDA priority review [12, 13]. Lack of consensus on diagnostic criteria and treatment approach presents significant challenges.

## Epidemiology

The incidence and mortality of TA-TMA varies widely due to heterogeneous diagnostic criteria, under-recognition, and the wide variety of treatments that are used. The incidence was estimated to be 8.2% in a comprehensive literature review published in 2004 aggregating the outcomes of 5423 allotransplant patients. One large review found a mortality rate of 75% with the majority of patients dying within 3 months of TA-TMA diagnosis [14]. Jodele et al. estimated the incidence of TA-TMA to be 39% in pediatric and young adult patients, with 20% having clinically meaningful disease however the majority of these patients underwent HSCT for nonmalignant diseases [15]. A recent, large review of nearly 2000 adult HSCT patients by Postalcioglu et al. using the City of Hope criteria (Table 1) identified 13% to have definite TMA and an additional 26% to have probable TMA [3]. Only 17% were clinically diagnosed, suggesting that this syndrome is under-recognized. In another large retrospective review done by Gavriilaki et al. of 758 HSCT recipients from a single institution over 27 years, the incidence of TA-TMA as defined by the International Working Group criteria was 15.5% [5]. Response rates vary from 25 to 93%, with lower response rates reported with plasma exchange and higher response rates with complement directed therapy. Mortality rates range from 40 to 84% [8, 10, 11, 16–18]. Higher response rates are reported with terminal complement directed therapy, but one study showed similar survival as compared with other therapies due to the increase in infectious complications that were not attributable to *N. meningitidis* [16].

**Table 1:** Diagnostic criteria for TA-TMA with comparison of proposed definitions and risk stratification.

Clinical or laboratory marker	CTN-TMA [19]	IWG-TMA [71]	City of Hope (COH)^f^ [76]	Overall-TMA, Cho et al. [50]	Joint Study Group, Uderzo et al. [73]	TA-TMA by Jodele et al.^g^ [15]
Schistocytosis	≥2/HPF	>4% (8/HPF)	Yes	≥2/HPF	>1–2/HPH	Yes
Negative direct and indirect Coombs test	Yes	–	–	Yes	Yes	–
Concurrent renal and/or neurologic dysfunction without other explanations^a^	Yes	–	SCr > 1.5 × baseline	–	Proteinuria and hypertension	Proteinuria and hypertension
Decrease in serum haptoglobin	–	Yes	–	Yes	–	–
De novo thrombocytopenia^b^	–	Yes	Yes	Yes	Yes	Yes
De novo anemia^c^	–	Yes	–	Yes	Yes	Yes
*Increase in serum LDH	*Yes*	*Yes*	*Yes*	*Yes*	*Yes*	*Yes*
*Hypertension^*d*^	*–*	*–*	*–*	*–*	*Yes*	*Yes*
*■Proteinuria^*e*^	*–*	*–*	*–*	*–*	*Yes*	*Yes*
*■Terminal complement activation (Elevated sC5b-9)*	*–*	*–*	*–*	*–*	*Yes*	*Yes*

*COH* City of Hope, *CTN* blood and marrow transplant clinical trials network, *HPF* high-power field, *IWG* International Working Group, *LDH* lactate dehydrogenase, *TA-TMA* transplant-associated thrombotic microangiopathy.

Italic region includes early screening and high-risk markers.

*Early markers of TMA (suspected TA-TMA) [15].

■ Markers of high-risk TMA (high-risk TMA) [15].

^a^Doubling of serum creatinine from baseline (baseline = creatinine before hydration and conditioning) or 50% decrease in creatinine clearance from baseline.

^b^Platelet count ≤50 × 10^9^/L or ≥50% or greater reduction from previous counts.

^c^Decrease in hemoglobin concentration or increased red blood cell transfusion requirement.

^d^A blood pressure ≥95% for age (<18 years old); ≥140/90 mmHg (≥18 years old); resistant to two or more antihypertensive agents.

^e^A random urinalysis protein concentration ≥30 mg/dL.

^f^Probable TA-TMA is defined as having three of the four criteria in addition to clinical TA-TMA diagnosis; definite TA-TMA is defined as having all four criteria.

^g^Diagnosis of TA-TMA based on microangiopathy confirmed by tissue biopsy or >4 diagnostic markers at the same time.

## Theoretical considerations

### Pathophysiology

TA-TMA is a syndrome of abnormal endothelial cell activation with features of thrombotic thrombocytopenic purpura (TTP) and hemolytic uremic syndrome (HUS) [19]. Defining features are endothelial injury and complement activation [15], without the depletion of ADAMTS13 as in primary TTP [20]. Various factors in the transplant process lead to development of endotheliitis and subsequent complement activation, formation of platelet rich thrombi, and microvascular hemolytic anemia that ultimately cascade into end organ dysfunction. A three hit hypothesis has been proposed: an underlying predisposition to complement activation or preexisting endothelial injury (hit 1), a toxic conditioning regimen causing endothelial injury (hit 2), followed by additional insults including medications, alloreactivity, and/or infections (hit 3). The accumulation of the three hits crosses a threshold triggering the activation of the complement cascade and microthrombi formation [21].

**Fig. 1:**
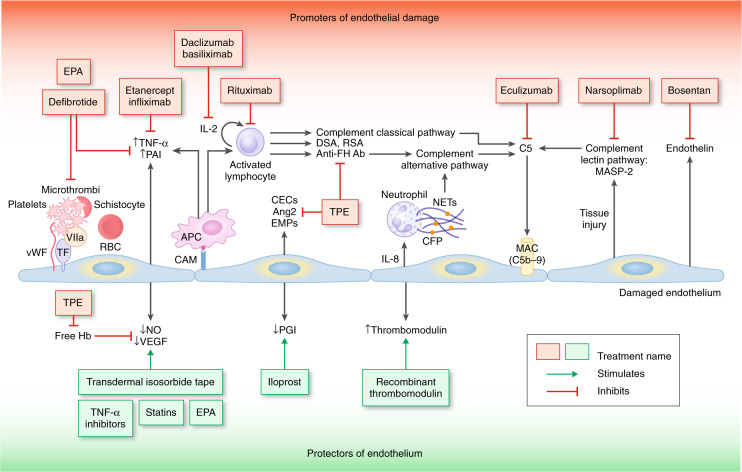
Pathophysiology of TA-TMA with the sites of action of proposed treatments. The illustration is separated into two halves. The upper half demonstrates the numerous mechanisms that promote endothelial damage and the treatments targeting these promoters of injury. The lower half represents the effect the damaged endothelium has on protective factors of the endothelium and the treatments, which enhance these cytoprotective factors. Therapies shown as potential treatment options but have not been widely or rigorously studied include EPA, TNF-α inhibitors (etanercept, infliximab), bosentan, transdermal isosorbide tape, statins, iloprost, and recombinant thrombomodulin. Adapted with copyright permission from Fig. 4 Khosla et al. [101]. Ang2 angiopoietin 2, APC antigen-presenting cell, CAM cell adhesion molecules, CEC circulating endothelial cell, CFP complement factor P, DSA donor-specific antibodies, EMP endothelial microparticles, EPA eicosapentaenoic acid, FH Factor H, Hb hemoglobin, IL interleukin, MAC membrane attack complex, MASP-2 mannose-binding protein-associated serine protease-2, NETs neutrophil extracellular traps, NO nitric oxide, PAI plasminogen-activator inhibitor, PGI2 prostacyclin, RBC red blood cell, TNF-α tumor necrosis factor alpha, RSA recipient-specific antibodies, TF tissue factor, TPE therapeutic plasma exchange, VIIa Factor VIIa, VEGF vascular endothelial growth factor, vWF von Willebrand factor.

Endothelial injury leads to an increase in proinflammatory cytokines, procoagulant factors, and soluble adhesion molecules, which promote further endothelial injury and initiate and propagate the complement cascade [22, 23]. Nitric oxide depletion diminishes the vasodilatory properties of the vessel, decreases the release of P-selectin and von Willebrand factor, and leads to platelet aggregation and subsequent microthrombi development (Fig. 1) [24, 25]. These findings are consistent but nonspecific; development of TA-TMA appears dependent on poorly understood host factors. Recent studies confirm the role of complement activation, indicated by significant increases in C3b and sC4b-9 [15, 26]. Patients who acquired variants of complement system regulatory proteins from their donors appear to be at increased risk for TA-TMA, suggesting that patients and donors could potentially be screened for these mutational variants prior to HSCT [27]. An association of HLA-DRB1*11 and idiopathic TTP has been recognized [28–30]. Among patients with TA-TMA, those with HLA-DRB1*11 had significantly better outcomes [31].

Recent work in pediatric patients with nonmalignant diseases suggests that neutrophil extracellular traps (NETs) may link endothelial injury and the subsequent activation of the complement cascade. The injured endothelium releases IL-8, causing neutrophil activation and release of NETs leading to complement activation and deposition of complement factor P, C5b-9, and formation of microthrombi [32].

### Risk factors

TA-TMA occurs after autologous and allogeneic HSCTs; incidence is much higher in the latter [33–35]. Risk factors for the development of TA-TMA include specific conditioning regimens, calcineurin (CNI) and mammalian target of rapamycin inhibitors (mTORi), the presence of graft versus host disease (GVHD), venous thromboembolic disease, ABO incompatibility [36], human leukocyte antigen mismatch [3], and infection [5, 37–39].

CNI and mTORi used for GVHD prophylaxis contribute to endothelial injury via direct cytotoxic damage, platelet aggregation, elevated von Willebrand factor and thrombomodulin, alterations in complement regulation, and decreased production of prostacyclin and nitric oxide [39, 40]. There are data to suggest that the risk of TA-TMA is further exacerbated in those receiving a CNI/mTORi in conjunction with busulfan and cyclophosphamide containing induction regimens [41].

An autopsy study revealed that patients with acute GVHD have a fourfold increase in TA-TMA compared to patients without it [42]. Some propose that TA-TMA is a form of endothelial GVHD [43, 44]; however, evidence to support a distinction includes the difference in management (increase of CNI versus withdrawal) as well as histologic findings on biopsy [33]. Development of TA-TMA in the setting of GVHD is attributed to endothelial injury secondary to increased circulating cytokines, decreased VEGF, activation of the coagulation cascade, and direct endothelial damage from cytotoxic donor T cells [43].

Infections associated with TA-TMA include aspergillus, cytomegalovirus, adenovirus, parvovirus B19, human herpes virus-6, and BK virus [14, 19, 34, 45–47]. Elevated levels of thrombomodulin, plasminogen-activator inhibitor type-1, IL-8, and interferon gamma have been observed in patients with viremia, likely playing a role in pathogenesis [23, 48].

Postalcioglu et al. described several risk factors associated with definite TMA defined by City of Hope criteria (Table 2), including HLA mismatched donor (HR 1.79; 95% CI 1.17–2.75; *p* = 0.007), sirolimus-containing GVHD prophylaxis (HR 1.73; 95% CI 1.29–2.34; *p* < 0.001), and myeloablative conditioning (HR 1.93; 95% CI 1.38–2.68; *p* < 0.001). CMV positive serostatus was a risk factor associated with probable TMA (HR 1.41; 95% CI 1.16–1.71; *p* < 0.001) [3].

**Table 2:** Clinical manifestations of TA-TMA by organ system with differential diagnoses and suggested evaluations.

Organ system	Clinical manifestations	Differential diagnoses	Diagnostic considerations (excluding tissue biopsy)
Kidney	Proteinuria, hypertension, acute, or chronic kidney injury	CNI, steroids (causing hypertension), nephrotoxic medications, acute cystitis, BK nephritis, and engraftment syndrome	—Urinalysis with urine culture
			—Urine protein/creatinine
			—Urine and blood PCR for BK virus
GI Tract	Severe abdominal pain, intestinal bleeding, diarrhea, vomiting, and ascites	GVHD, hepatic SOS, infectious colitis, drug-induced colitis, and engraftment syndrome	—Liver ultrasound
			—Coagulation studies
			—Liver blood tests (total and direct bilirubin, albumin, alkaline phosphatase, AST, ALT)
			—Amylase, lipase
			—Viral serologies if not already performed (including hepatitis, CMV, HSV)
			—Consider stool culture
			—Consider skin biopsy if question of GVHD
CNS	Headache, seizures, confusion, and hallucinations	Encephalitis, fludarabine toxicity, delirium, psychiatric disorder, and engraftment syndrome	—Brain MRI
			—Viral serologies
			—Nutritional evaluation including thiamine
Cardio-pulmonary/polyserositis	Pulmonary hypertension, pleural effusion, and refractory pericardial effusion	Infection, pulmonary edema, engraftment syndrome, GVHD, PVOD, drug toxicity, radiation pneumonitis, connective tissue disease, idiopathic pneumonia syndrome, and ACS	—Chest imaging
			—Echocardiogram
			—Bronchoscopy
			—Pulmonary function testing
			—ECG, troponin
			—Skin biopsy

*ALT* alanine transaminase, *AST* aspartate transaminase, *ACS* acute coronary syndrome, CMV cytomegalovirus, *CNI* calcineurin inhibitor, *ECG* electrocardiogram, *GI* gastrointestinal, *GVHD* graft versus host disease, *HSV* herpes simplex virus, *PCR* polymerase chain reaction, *PVOD* pulmonary veno-occlusive disease, *MRI* magnetic resonance imaging, *SOS* sinusoidal obstruction syndrome, *TA-TMA* transplant-associated thrombotic microangiopathy.

## Clinical considerations

### Clinical manifestations

While primarily associated with kidney involvement, endothelial damage in TA-TMA can occur in multiple organs leading to intestinal TMA (iTMA), pulmonary hypertension, posterior reversible encephalopathy syndrome, and polyserositis. A high index of clinical suspicion is needed as presentation is similar to that of other post-HSCT complications. Patients may also present with multiple organ dysfunction syndrome (MODS) which is associated with high morbidity and mortality [7, 49]. We highlight organ-specific manifestations followed by a critical evaluation of proposed diagnostic criteria.

#### Kidney

The kidney is typically the primary site of microangiopathy, causing proteinuria, acute kidney injury, hypertension, development of chronic kidney disease, or end stage renal disease. Kidney involvement is a significant prognostic factor associated with poor survival and highlights the need for early recognition [3, 50]. The diagnosis of kidney involvement is ideally confirmed by renal biopsy demonstrating small vessel injury with renal arterioles showing intraluminal microthrombi and fibrin, endothelial cell separation, and widening of the subendothelial space. Glomerular changes include mesangiolysis and double contours of the basement membrane. Intraluminal schistocytes are also often observed in the affected glomerular capillaries [51]. C4d deposition in renal arterioles has been reported [33, 52]. Unfortunately, tissue diagnosis may be precluded by the risks of HSCT-associated co-morbidities and thrombocytopenia, emphasizing the need for other markers of renal involvement.

Blood pressure is easily measured and hypertension is a clinically important indicator of renal involvement [53]. Jodele et al. prospectively studied 100 children and young adults (mean age 8.3 years), undergoing HSCT. In the thirty-nine subjects who met criteria for TA-TMA, hypertension and proteinuria along with increased lactate dehydrogenase (LDH) occurred 10–14 days prior to TMA diagnosis [15]. Hypertension is a common complication of transplantation; however, several reviews have recommended evaluation for TA-TMA in patients who require extensive treatment for hypertension (i.e., more than two agents or treatment in excess of that expected) [49, 54].

Proteinuria is an important indicator of renal dysfunction in TA-TMA and is both an early sign of renal endothelial injury and a marker of severe disease and increased mortality [15, 35, 44, 55]. Proteinuria is easily identified on routine urinalysis followed by spot urine protein-to-creatinine ratio. Similar to routine blood pressure checks, regular urine monitoring is an inexpensive diagnostic tool [49].

Unlike hypertension and proteinuria, elevated creatinine is generally a late feature of TA-TMA concerning for development of irreversible kidney injury [15, 50]. Measurement of serum creatinine and creatinine-based GFR estimates are further limited by low sensitivity in the pediatric, elderly, and malnourished populations [56]. In children, Laskin et al. suggest that cystatin C may be a better indicator of early renal dysfunction [57, 58]. However, evidence of renal involvement, defined as a doubling of serum creatinine or 50% decrease in creatinine clearance from baseline, is a poor prognostic marker [50].

#### Gastrointestinal tract

Histologic studies show both intrarenal and extrarenal microthrombi suggesting that TA-TMA is a multisystem disorder [51, 59]. iTMA is an increasingly recognized manifestation and is associated with high-risk features [15, 59–62]. iTMA presents with a constellation of signs and symptoms including severe abdominal pain, diarrhea, vomiting, ascites, and intestinal bleeding, which are similar to that of GI GVHD, infectious colitis, or drug-induced colitis. Diagnosis is confirmed on examination of submucosal vasculature. Lack of histologic criteria to differentiate iTMA from GVHD also confounds diagnosis [63].

In a large retrospective single-center analysis, Inamoto et al. found that 92% of adult patients who had a post-transplant colonoscopy for severe diarrhea attributed to GVHD had histologic signs of iTMA; only 30% had evidence of concomitant GVHD. iTMA was a major cause of NRM at 57% in patients without resolution of diarrhea [60]. An autopsy series performed by Yamada et al. showed histopathologic evidence of TA-TMA in 23%. The kidney was most frequently affected (61%) followed by intestines (53%), most commonly the right colon and ileum. Overlap of renal and iTMA was found in only 13% of cases. Patients in the iTMA group had a higher frequency of intestinal GVHD than those of the renal TA-TMA group (80% vs. 22%) suggesting that compared to renal TA-TMA, intestinal GVHD may be more closely associated with iTMA [51].

These studies are limited by their retrospective nature with risk of selection bias and lack of consensus criteria. El-Bietar et al. proposed eight histologic markers of bowel vascular injury in patients with high-risk systemic TMA based on a pediatric population; the development of iTMA in the absence of systemic high-risk TMA was not reported [61]. Application of these criteria to a larger cohort of mostly adult patients (age range 19–71 years versus 1–29 years) did not demonstrate diagnostic sensitivity or specificity; however, the authors suggest that findings of microthrombi, schistocytes, and mucosal hemorrhage should raise suspicion for the presence of concomitant TA-TMA [63]. A high index of suspicion is required to identify iTMA as it can present in the setting of systemic TA-TMA or as an isolated entity that mimics GVHD.

#### Central nervous system

Neurologic findings are less prominent in TA-TMA than in de novo TTP [50, 64]. Symptoms include headache, seizures, confusion, and hallucinations. Posterior reversible encephalopathy syndrome has been described in pediatric patients due to uncontrolled TA-TMA associated hypertension [49, 65]. CNS involvement is reported as a significant unfavorable predictor of mortality [5].

#### Serosal surfaces

Traditionally, significant cardiac and pulmonary vascular endothelial toxicities after HSCT were attributed to chronic GVHD or chemotherapy and radiation effects [66, 67]. However, polyserositis has been described in TA-TMA, presenting as refractory pericardial effusion, pleural effusion, and ascites without overall generalized edema [49]. In pediatric studies the incidence of pericardial effusions in TA-TMA is higher than in the overall HSCT population. Despite surgical drainage, effusions often recur until the serositis resolves [6, 66, 68]. In the absence of GVHD involvement in other organs, the diagnosis of TA-TMA should be considered for recurrent pleural and pericardial effusions.

#### Cardio-pulmonary

Pulmonary vascular involvement in TA-TMA can manifest with findings of respiratory distress and hypoxemia related to pulmonary hypertension. While uncommon, it should be suspected in patients with unexplained hypoxemia given the associated high mortality [69]. The gold standard for diagnosis is cardiac catheterization, but invasive testing is often not possible due to HSCT-associated co-morbidities [70]. In a prospective echocardiographic screening study looking at pediatric patients, Dandoy et al. found that elevated right ventricular pressure on Day +7 after HSCT (*n* = 13, 13%) was significantly associated with development of TA-TMA, suggesting the potential utility of echocardiography as a screening tool for early vascular injury associated with TA-TMA [6].

#### MODS

There is a spectrum of severity in TA-TMA ranging from self-limited disease, chronic organ dysfunction, to high-risk systemic MODS requiring intensive care support [7, 15]. Systemic TA-TMA is associated with significant morbidity and mortality and underscores the need for preventive measures and early multidisciplinary approach to diagnosis [5]. Table 2 highlights the varied presentations of TA-TMA by organ system, differential diagnoses, and suggested evaluations.

### Diagnostic criteria: a critical evaluation

The reported incidence of TMA after HSCT varies widely due to a lack of consensus diagnostic criteria. Moreover, the clinical features of TA-TMA including organ dysfunction and cytopenias are common after HSCT and may result from drug effect, infection, GVHD, or other vascular endothelial damage syndromes. While there is a large variability in published median time to onset, timing of symptom onset may provide some clues. A large Greek HSCT study showed TA-TMA onset at a median of 86 days, with a range from 9 to 721 days [5]. In the autopsy study done by Yamada et al. median onset of TA-TMA was 57 days (17–472) [51]. Most cases occurred before 100 days which may help distinguish it from other post-transplant complications.

Unfortunately, there is significant diversity among the proposed diagnostic criteria, of which most are based upon retrospective analysis (Table 1). Initially two proposed consensus criteria were developed for TA-TMA: Bone Marrow Transplant Clinical Trials Network (CTN-TMA, 2005) and International Working Group of the European Group for Blood and Bone Marrow Transplantation (IWG-TMA, 2007) [19, 71]. Limitations which lower diagnostic sensitivity were described in a retrospective analysis. CTN-TMA criteria require concurrent renal and/or neurologic dysfunction and IWG-TMA criteria requires >4% schistocytes in blood [72]. Additionally, haptoglobin can be an unreliable marker of the presence or extent of hemolysis since it is also an acute phase reactant [73]. While serial measurement of schistocytes on the peripheral blood smear has traditionally been thought the defining feature for microangiopathy, schistocytosis is not a predictive factor for the early diagnosis of TMA [15, 74, 75].

The inclusion of acute renal or neurologic impairment as a diagnostic requirement is controversial as these conditions are frequently not present at the onset of TA-TMA [46, 64]. With these limitations in mind and in an attempt to expedite early diagnosis in TA-TMA, Cho et al. proposed a novel set of diagnostic criteria in 2010, termed overall-TMA (O-TMA), which does not require renal or neurologic findings [50]. The common elements among these three criteria are elevated LDH and the presence of schistocytes on the peripheral blood smear. Two additional sets of diagnostic criteria have been proposed by the Joint Study Group and City of Hope and are summarized in Table 2 [73, 76]. There is a clear need for unified objective and organ-specific criteria to assist in the timely recognition of TMA and for use in future clinical trials.

#### Screening and evaluation

Jodele et al. proposed a new set of diagnostic criteria based on her prospective trial that emphasized early diagnosis and risk stratification. The resulting proposal incorporates many recent insights into the pathophysiology of TA-TMA, including the first use of a complement marker (serum C5b-9). Hypertension, proteinuria, and an increased LDH were present 10–14 days prior to TMA diagnosis suggesting their potential use as screening markers for TA-TMA [15].

Workup should exclude other diagnoses such as medication toxicities, infectious complications, GVHD, and other vascular endothelial syndromes, as shown in Table 2, along with DIC, TTP, and Coombs-positive hemolytic anemia. Normal coagulation studies, ADAMTS13 activity >10%, and negative direct antiglobulin test suggest TA-TMA as the diagnosis if other criteria are also met. Of note, the PLASMIC score has been shown to be a predictor of severe ADAMTS13 deficiency in idiopathic TTP but not in the post-HSCT setting [77].

#### High-risk TA-TMA

High-risk TA-TMA was initially defined as presence of proteinuria and evidence of terminal complement activation (elevated serum C5b-9) and was associated with an 84% NRM at 1 year after HSCT. TA-TMA patients without these features had a survival rate of 100% [15]. In a recent large cohort of pediatric patients, Jodele et al. identified 31% with TA-TMA of which 36% were deemed to have high-risk disease, revised to also include MODS. Specifically, high-risk TA-TMA was defined as nephrotic range proteinuria (proteinuria ≥ 30 mg/dL × 2 or random urine protein/creatinine ratio ≥ 2 mg/mg) and elevated sC5b-9 (>244 ng/mL) at time of TA-TMA diagnosis or presence of one of these two high-risk laboratory features with clinical evidence of MODS. In these patients, eculizumab was shown to be an effective therapeutic strategy with improved 1-year post-HSCT survival, although subjects with higher sC5b-9 level at the start of therapy were less likely to respond to treatment and required more drug doses [9].

### Treatment

**Fig. 2:**
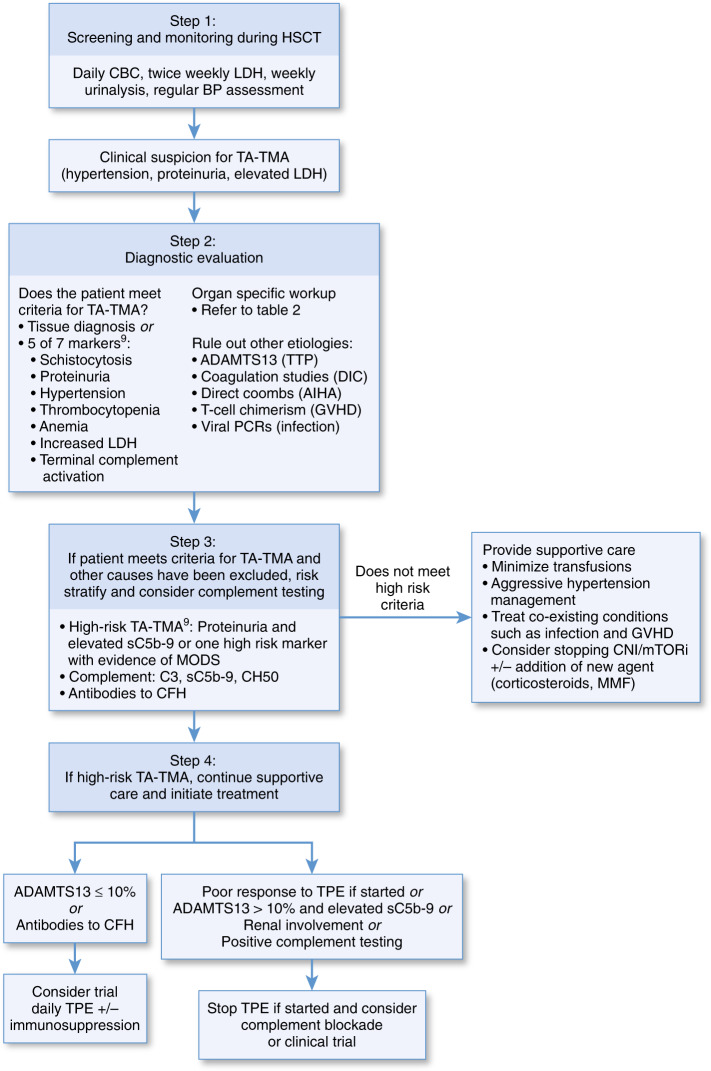
Proposed TA-TMA diagnostic and treatment algorithm. AIHA autoimmune hemolytic anemia, BP blood pressure, CBC complete blood count, CFH complement factor H, CNI calcineurin inhibitor, DIC disseminated intravascular coagulation, GVHD graft vs. host disease HSCT hematopoietic stem cell transplantation, LDH lactate dehydrogenase, MMF mycophenolate mofetil, MODS multiple organ dysfunction syndrome, mTORi mammalian target of rapamycin inhibitor, PCR polymerase chain reaction, TA-TMA transplant-associated thrombotic microangiopathy, TPE therapeutic plasma exchange, TTP thrombotic thrombocytopenic purpura.

In recent years, the approach to treatment of TA-TMA has evolved based on increased understanding of underlying pathophysiology. Preventative measures such as avoidance of endothelial toxins, avoidance of infection, and optimization of conditioning regimens may minimize endothelial injury. Aggressive supportive care including minimizing transfusions, aggressive hypertension management, and treatment of any underlying infection are central to TA-TMA treatment. Additional approaches include withdrawal of CNI/mTORi, therapeutic plasma exchange (TPE), rituximab, defibrotide, and eculizumab (Fig. 1). Clinical trials have explored novel treatment approaches with various complement directed therapies including mannan-binding lectin-associated serine protease-2 inhibition (MASP-2) and second generation anti-C5 directed therapy. Prompt recognition and early initiation of treatment may lead to improved outcomes as suggested by recent literature [9, 78]. We propose a clinically focused diagnostic and treatment algorithm (Fig. 2), based on the literature reviewed here.

#### Withdrawal of CNI/mTORi

As CNI can contribute to the development of TA-TMA, they are commonly withdrawn upon recognition of the syndrome [19]; some centers even report addition of CYP3A4 targeted agents to induce clearance [4]. However, benefit from these interventions has not been rigorously proven [46]. Cyclosporine levels have not been correlated with TA-TMA and supratherapeutic tacrolimus levels at the time of TA-TMA diagnosis have not been correlated with poorer outcomes [42, 79]. Withdrawal of CNI should be performed cautiously, as exacerbation of GVHD could potentiate TA-TMA. As an alternative to CNI and mTORi, Wolff et al. explored the use of the IL-2 inhibitor daclizumab in patients with GVHD and TA-TMA. In this 13-patient cohort, 9 patients achieved complete remission of TA-TMA, two had stable disease, and one did not respond. Mortality from acute GVHD in this group was high despite the use of daclizumab. This data suggests that in select cases, it may be reasonable to utilize an IL-2 receptor antagonist such as basiliximab (as daclizumab is no longer available) for prevention or treatment of GVHD in the setting of concomitant TA-TMA [80].

#### Therapeutic plasma exchange

Prior to the elucidation of dysregulated complement as a driver in the development of TA-TMA, TPE was a mainstay of treatment. However, with increased understanding of the pathophysiology of TA-TMA and recognition that ADAMTS13 levels in TA-TMA are normal or only slightly decreased, treatment strategies have shifted away from this approach. The only prospective evaluation of TPE in TA-TMA reported a response rate of 64% [36]. In a single-center study comprised of 66 patients who developed TA-TMA after HSCT while on tacrolimus, of the 63 who received TPE, 60% experienced response. Six month survival was 0% in nonresponders and 50% in responders [79]. A recent retrospective study of 15 patients showed that treatment with TPE did not prevent development of chronic kidney disease in patients with TA-TMA [81].

When patients have documented factor H autoantibodies, TPE may be useful [78]. Timing may also be an important element influencing efficacy, as one study in pediatric patients demonstrated that earlier initiation led to higher response rates [78]. As TPE is not without adverse effects and in fact, some studies have shown worse outcomes with the use of TPE [19, 82], it should be used cautiously in TA-TMA, only if other complement directed therapies are not available or if the clinical scenario suggests the presence of autoantibodies and therapy can be initiated early in the disease course. According to the American Society of Apheresis guidelines, the role of TPE for TA-TMA is a weak, grade 2C recommendation, due to low quality evidence [83].

#### Rituximab

The anti-CD20 antibody rituximab, with its mechanism of antibody depletion and immune regulation, is commonly and successfully used in primary TTP in conjunction with TPE. Evidence for use in TA-TMA is limited to single patient case reports [84–92]. It can be considered in conjunction with TPE on a case-by-case basis.

#### Defibrotide

Because of defibrotide’s protective effects on the endothelium and its ability to restore thrombotic-fibrinolytic homeostasis in small vessels, it has been employed to treat TA-TMA [93]. In a retrospective study done in Spain of 17 adult patients with TA-TMA, defibrotide was given as monotherapy in 5, and in combination with other therapies in the remainder. The drug was generally well tolerated and complete resolution was observed in 65% with an OS of 59% [18]. Yeates et al. presented a series of 17 defibrotide treated patients with resolution of TA-TMA achieved in 76% [94]. Bohl et al. observed that patients treated with defibrotide with or without TPE and/or rituximab had a response rate of 61% [16]. These findings support efficacy of defibrotide and evaluation in prospective trials is warranted.

#### Complement directed therapy

Eculizumab is a humanized monoclonal anti-C5 antibody that blocks the terminal complement pathway and prevents membrane attack complex formation. Published literature on the use of eculizumab in TA-TMA is limited to small retrospective cohorts with one exception. The studies include a wide range of patients who developed TA-TMA after transplantation for malignant as well as nonmalignant indications. Many of these studies involved patients previously treated with TPE and rituximab as well as withdrawal of CNI/mTORi. Time to initiation varied widely, and while most dosing strategies adhered to the complement mediated HUS (CM-HUS) dosing of 900 mg weekly for 4 weeks followed by 1200 mg biweekly, some studies dosed based on eculizumab trough values and complement levels. Overall, response rates were reported in 50 to 93% and OS rates ranged from 33 to 60% [8, 10, 11, 16, 17, 95, 96]. One group observed that the fastest and most complete responses were seen when treatment was initiated early and when early and sustained therapeutic levels of eculizumab and complement blockade were achieved [95]. Jodele et al. recently published a prospective study on the use of eculizumab in 64 high-risk TA-TMA pediatric patients under age 18 years. At 1 year, response rate was 64% and OS was 66%. Of those who died, 2 had resolution of TA-TMA before death but the remaining 27 had signs of active TMA at death [9]. It should be noted that eculizumab was dynamically dosed based on eculizumab trough and CH50 levels.

Based on these studies, eculizumab should be considered a front-line therapy for TA-TMA in patients with evidence of complement activation. Preferred dosing schedules may differ from that of typical dosing for CM-HUS, although dynamic dosing has not been evaluated in adults. A 4–6 week period of induction therapy during which eculizumab trough levels and CH50 levels can be followed with additional dosing as necessary to maintain therapeutic effect appears reasonable [9, 95, 97]. Unlike CM-HUS, patients who develop TA-TMA may not require life-long eculizumab therapy, since the stimulus for endothelial injury and complement dysregulation in most cases is temporary. Successful cessation of therapy was demonstrated in several studies, with only one documented recurrence that responded to repeat dosing [9]. Consideration of eculizumab cessation should only occur after full resolution of hematologic TMA markers and full complement blockade as determined by a low CH50 level. Administration of meningococcal vaccination and primary prophylaxis against *Neisseria meningitidis* are recommended with use of eculizumab [98].

It should be noted that eculizumab is not FDA approved for TA-TMA. Although current use for complement directed therapy is FDA approved for microangiopathic hemolytic anemia, specifically atypical HUS and paroxysmal nocturnal hemoglobinuria, its use in TA-TMA has been explored in pharmaceutical industry-initiated research. Because of the role of complement in TA-TMA, it was logical to investigate the use of these agents as adjunctive therapies. Despite encouraging initial findings with eculizumab, a study of an anti-C5 compound LFG316 was stopped early after findings in 7 patients demonstrated low likelihood of clinical benefit (NCT02763644) [99]. Further large prospective trials using eculizumab in adults with TA-TMA are needed.

#### MASP-2 inhibition

The MASP-2 inhibitor narsoplimab (OMS721) is a novel selective complement inhibitor thought to block complement activation and endothelial injury without the immunologic dysfunction observed with broad complement inhibition. It targets mannan-binding lectin-associated serine protease-2, the effector enzyme of the lectin pathway in the complement system. There is a theoretical benefit of selectively targeting only one of the principal complement pathways. By leaving intact the respective functions of the other pathways of innate immunity, widespread damage may be avoided [13]. As presented at the European Hematology Association conference in 2018, in the 19 narsoplimab treated patients (median age 50, 17 with underlying malignant conditions, all had undergone allogeneic HSCT), improvement was seen in platelet count, LDH, and haptoglobin. Notably, no improvement in creatinine was seen. Compared to historical controls, median OS at 347 days (95% CI 79-NE, *p* < 0.0001) and survival at day 100 were significantly improved (53% vs. 10%, *p* = 0.0002). Serious adverse events were GVHD, neutropenic sepsis, and acute renal failure [100]. This was a small study, limited by lack of data regarding the use of CNI or MTORi and the specific dosing regimen used. Given the compelling improvement in OS and disease markers, narsoplimab (OMS721) is now under priority review by the FDA.

## Conclusions and future directions

TA-TMA remains an elusive diagnosis with profound implications on morbidity and mortality. iTMA has recently been recognized as a distinct clinical entity along the spectrum of TA-TMA. Substantial recent progress has been made in understanding of pathophysiologic mechanisms and discovery of potential effective treatments. Nonetheless, development of TA-TMA still confers high-risk of progression to MODS and death, and early detection and intervention are critical. Hypertension and proteinuria are easily obtainable measures that may be early indicators of the disease process. Elucidation of the role of complement in development of TA-TMA has shifted biologic rationale of treatment toward terminal complement inhibition with eculizumab and MASP-2 inhibition with narsoplimab (OMS721). The prospective data on the use of eculizumab is limited to the pediatric population. Retrospective data in the use of defibrotide in TA-TMA is promising and further studies are warranted. As proposed by the Joint Study Group, prospective controlled studies investigating defibrotide and eculizumab should be conducted. Risk stratification that incorporates markers of complement activation and MODS may facilitate prompt recognition and initiation of targeted therapies. We have proposed a practical diagnostic and treatment approach for suspected TA-TMA (Fig. 2).

A wealth of new studies in pathophysiology and treatment in recent years adds to our collective understanding of TA-TMA, but nearly all have been performed in pediatric patients, and application to the adult population remains unproven. Notable differences in underlying co-morbidities, disease process, and response between adults and children have the potential to confound diagnosis and diminish treatment efficacy. Nonetheless, prospective, multi-institution studies should focus on the HSCT adult population as data are sorely lacking. As TA-TMA is rare, large randomized trials may not be feasible, and prospective controlled studies would benefit from cooperative group involvement. Additionally, further optimization and validation of scoring systems with particular attention to the adult population are needed and should be incorporated into future studies. Establishment and widespread use of consensus criteria for diagnosis of TA-TMA will be crucial in designing multicenter treatment trials in adult HSCT patients with the goal to minimize TA-TMA related morbidity and mortality.
